# Prevalence and characteristics of hypoxic hepatitis in the largest single-centre cohort of avian influenza A(H7N9) virus-infected patients with severe liver impairment in the intensive care unit

**DOI:** 10.1038/emi.2016.1

**Published:** 2016-01-06

**Authors:** YiMin Zhang, JiMin Liu, Liang Yu, Ning Zhou, Wei Ding, ShuFa Zheng, Ding Shi, LanJuan Li

**Affiliations:** 1 State Key Laboratory for Diagnosis and Treatment of Infectious Diseases, Collaborative Innovation Center for Diagnosis and Treatment of Infectious Diseases, the First Affiliated Hospital, College of Medicine, Zhejiang University, Hangzhou 310003, Zhejiang Province, China; 2 Department of Pathology and Molecular Medicine, Faculty of Health Sciences, McMaster University, Hamilton L8S 4L8 ON, Canada; 3 Department of Pathology, the First Affiliated Hospital, College of Medicine, Zhejiang University, Hangzhou 310003, Zhejiang Province, China

**Keywords:** avian influenza A(H7N9), hypoxic hepatitis, liver impairment, prevalence

## Abstract

Avian influenza A(H7N9) virus (A(H7N9)) emerged in February 2013. Liver impairment of unknown cause is present in 29% of patients with A(H7N9) infection, some of whom experience severe liver injury. Hypoxic hepatitis (HH) is a type of acute severe liver injury characterized by an abrupt, massive increase in serum aminotransferases resulting from anoxic centrilobular necrosis of liver cells. In the intensive care unit (ICU), the prevalence of HH is ∼1%–2%. Here, we report a 1.8% (2/112) incidence of HH in the largest single-centre cohort of ICU patients with A(H7N9) infection. Both HH patients presented with multiple organ failure (MOF) involving respiratory, cardiac, circulatory and renal failure and had a history of chronic heart disease. On admission, severe liver impairment was found. Peak alanine aminotransferase (ALT) and aspartate aminotransferase (AST) values were 937 and 1281 U/L, and 3117 and 3029 U/L, respectively, in the two patients. Unfortunately, both patients died due to deterioration of MOF. A post-mortem biopsy in case 1 confirmed the presence of centrilobular necrosis of the liver, and real-time reverse transcription polymerase chain reaction of A(H7N9)-specific genes was negative, which excluded A(H7N9)-related hepatitis. The incidence of HH in A(H7N9) patients is similar to that in ICU patients with other aetiologies. It seems that patients with A(H7N9) infection and a history of chronic heart disease with a low left ventricular ejection fraction on admission are susceptible to HH, which presents as a marked elevation in ALT at the time of admission.

## Introduction

Avian influenza A(H7N9) virus (A(H7N9)) emerged in February 2013 and infected 571 patients according to the latest World Health Organization (WHO) report, updated on 23 February 2015.^1,2,3^ The mortality rate of A(H7N9) infection is 37.1% (212/571).^2^ Liver derangement commonly occurs in A(H7N9)-infected patients.^4,5,6^ Indeed, these patients can suffer from severe liver injury; however, the underlying reason remains unclear.

The causes of severe acute liver impairment include hepatotropic virus infection, drug-induced liver injury (DILI), ischaemic liver injury and liver trauma. Thus, whether the novel A(H7N9) virus is hepatotropic and plays an important role in the dramatic liver injury sometimes seen in patients with severe liver impairment warrants investigation. Furthermore, it is also necessary to identify other causes of severe liver impairment if A(H7N9)-related hepatitis is excluded in these patients.

Hypoxic hepatitis (HH) is characterized as a massive but transient increase in serum alanine aminotransferase (ALT) activity secondary to anoxic necrosis of centrilobular liver cells.^7^ The prevalence of HH in intensive care unit (ICU) patients is 1%–2%.^7^ HH is one cause of acute liver injury in patients with respiratory failure.^7,8^ Severe A(H7N9) infection always leads to respiratory failure, which reduces oxygen supply to the liver.^9^ Hence, HH is likely one possible cause of severe liver impairment in A(H7N9)-infected patients with respiratory failure. HH patients are not always on hepatology wards, which may complicate its recognition. To the best of our knowledge, there has been no report of HH in patients infected with A(H7N9) or any other type of avian influenza virus. However, the overall in-hospital mortality rate of HH can be as high as 45%–72% due to accompanying multiple organ failure (MOF).^10,11,12,13^ Because of this high mortality rate, early diagnosis and treatment of HH are necessary to ensure a good outcome.^11^

From March 2013 to February 2015, 112 patients with A(H7N9) infection were admitted to the ICU of the First Affiliated Hospital, Zhejiang University School of Medicine which represents the largest single-centre cohort of patients with A(H7N9) infection worldwide. We report here the prevalence of HH in this single-centre cohort. The clinical characteristics of A(H7N9)-infected patients with HH are described. Post-mortem biopsies were obtained from one fatal case of HH with A(H7N9) infection. The pathological features were reported, and A(H7N9)-related hepatitis was excluded based on a negative viral real-time reverse transcription polymerase chain reaction (RT-PCR) assay of liver tissue.

## Materials and methods

### Patients and clinical investigation

A total of 112 patients with avian flu H7N9 infection admitted to the ICU at the First Affiliated Hospital, Zhejiang University School of Medicine from March 2013 to February 2015 were screened. This study was approved by the Ethics Committee of the First Affiliated Hospital. Liver function was tested daily for the first week and at least twice during the remainder of the hospital stay. The presence of respiratory, cardiac, renal or circulatory failure was recorded. Serologic testing for viral hepatitis A, B, C, D, E, Epstein–Barr virus and cytomegalovirus was performed. Serum autoantibodies were also assayed. Liver ultrasound, echocardiogram and chest radiography were performed. All relevant medical information, such as past history, recent contact with live poultry, coagulation function and medications taken during the hospital stay, was recorded.

### HH criteria

Patients who met all of the following criteria were diagnosed as having HH according to previous reports^7,8,12^: (i) a massive but transient elevated ALT level (more than 20-fold the upper limit of normal (ULN)), (ii) the presence of respiratory, cardiac or circulatory failure and (iii) exclusion of other causes of liver injury.

### Post-mortem biopsy, histological examination and RT-PCR

A limited post-mortem biopsy that included the liver, lung and kidney was performed in one confirmed fatal case of HH with A(H7N9) infection (case 1). Written consent was obtained from the relatives of the patient prior to the biopsy. Two post-mortem biopsy cores from the liver and one biopsy from each lung and kidney were taken according to institutional protocols. One biopsy core from each organ was fixed in 10% formalin for histopathological examination by haematoxylin and eosin staining. The second liver biopsy core was directly homogenized and subjected to real-time RT-PCR for A(H7N9)-specific genes as reported previously.^14^ The primers and probes used are listed in Table 1.

### Statistical analysis

Statistical analysis was performed using the SPSS software package (version 17.0, Chicago, IL, USA). Non-parametric variables are presented as *n* (%). Parametric variables are presented as means ± SD and were compared by *t*-test. A *P-*value < 0.05 (two-tailed) was considered to indicate significance.

## Results

The 112 patients in the ICU with A(H7N9) infection were predominantly male (*n* = 75, 67.0%), aged 14–65 years (*n* = 78, 69.6%) (Table 2). Their underlying medical conditions, described in Table 2, included hypertension (*n* = 54, 48.2%), coronary heart disease (*n* = 12, 10.7%), chronic obstructive pulmonary disease (*n* = 6, 5.4%), cerebrovascular disease (*n* = 5, 4.5%), chronic liver disease (*n* = 7, 6.3%), chronic renal disease (*n* = 4, 3.6%), diabetes mellitus (*n* = 17, 15.2%), rheumatoid arthritis (*n* = 3, 2.7%) and cancer (*n* = 7, 6.3%). The proportion of patients with liver impairment was lower on admission than during the hospital stay (*n* = 27 (24.1%) vs. *n* = 51 (45.5%), *P* < 0.001). Antiviral therapy was performed in all 112 patients, while antibiotic therapy was performed in 91 (81.3%) and glucocorticoid therapy in 63 (56.2%) (Table 2).

An ALT level of >20-fold the ULN was found in only two patients, not only on admission but also during the hospital stay. The extent of ALT elevation was considerably higher in HH patients than in non-HH patients with liver injury (on admission, 1079.50 ± 41.72 U/L vs. 79.50 ± 56.34 U/L, *P* < 0.0001; hospital stay, 1109.00 ± 243.24 U/L vs. 102.90 ± 86.11 U/L, *P* < 0.0001) (Table 2). These two cases were diagnosed as HH. The incidence of HH in our single-centre cohort of ICU patients with A(H7N9) infection was 1.8% (2/112). Both cases exhibited respiratory, renal, circulatory and cardiac failure. In addition, viral hepatitis and autoimmune diseases were excluded based on the negative results of serum marker tests. DILI was excluded based on no history of drug intake (such as acetaminophen, Chinese herbs, etc.) suspected to induce DILI prior to admission. Liver ultrasound excluded surgical biliary tract diseases and space-occupying lesions. Details of the two patients are reported below.

### Case 1

An 86-year-old male was admitted to the ICU with a 5-day history of shortness of breath and sudden deterioration with a fever of 38.8 °C prior to admission. On admission, the patient exhibited ventricular tachycardia and was in atrial fibrillation (130 bpm), and had tachypnoea (33/min), oliguria and a low oxygen index (PaO_2_/FiO_2_) (∼60 mmHg). His mean arterial pressure was maintained at 70 mmHg by norepinephrine administration at a dose of 0.67 μg/kg·min.

**Figure 1:**
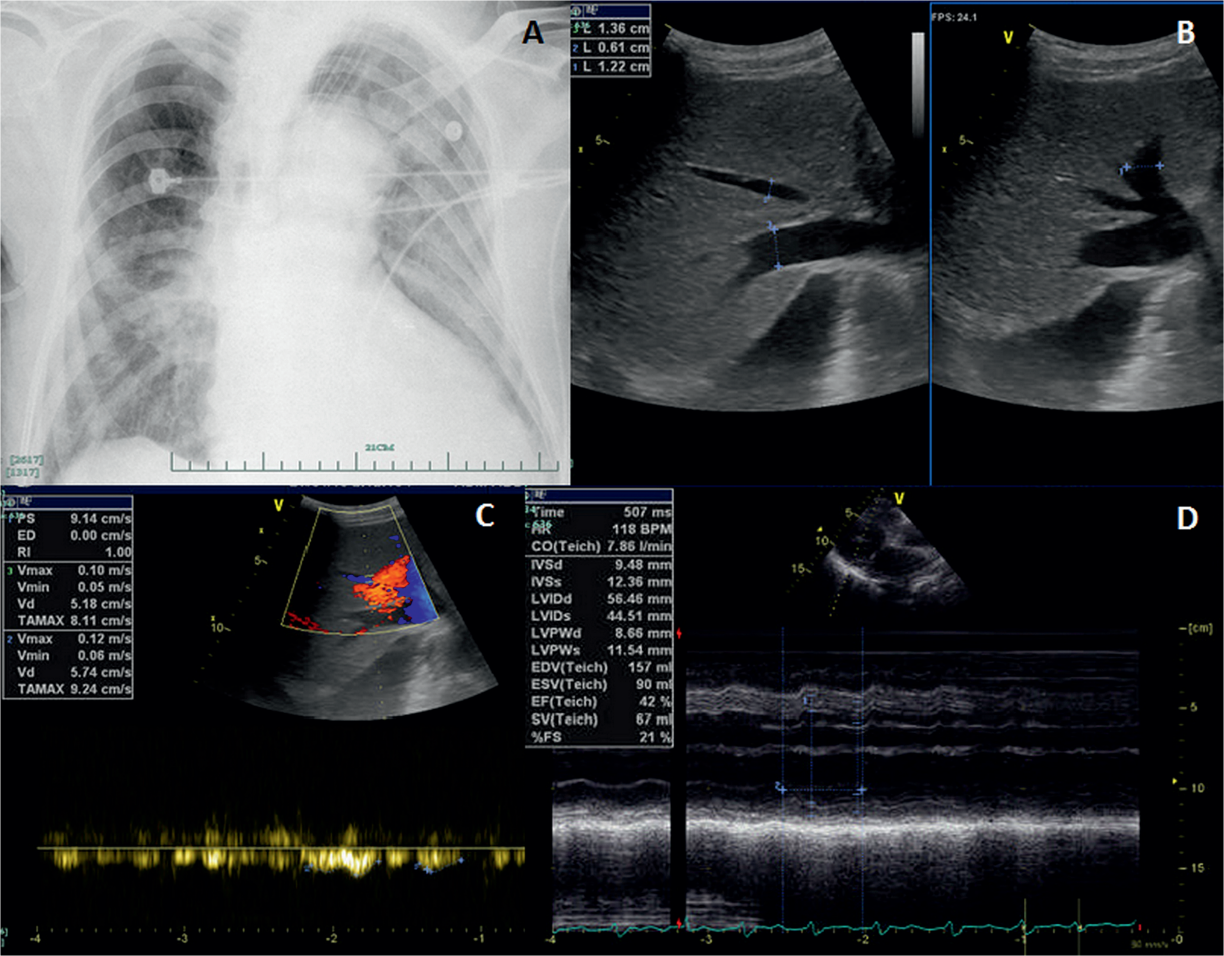
Radiographic and ultrasound findings in case 1. (**A**) Chest radiograph at the time of admission showed bilateral pulmonary infiltration with consolidation. (**B**) Ultrasound image showed a dilated hepatic vein (left branch, 1.22 cm; middle branch, 0.61 cm and right branch, 1.36 cm). (**C**) Ultrasound image showed slowed hepatic vein flow (middle branch, 9.14 mm/s). (**D**) Echocardiogram showed a dilated left ventricle (LVIDd 56.46 mm) and cardiac failure (LVEF 42%) with weakened left-ventricular wall motion. LVEF, left ventricular ejection fraction; LVIDd, left ventricular internal diameter at end-diastole.

The patient had a history of contact with live poultry seven days before admission. Chest radiography showed bilateral pulmonary infiltrates with consolidation at admission (Figure 1A). Respiratory secretions were positive for A(H7N9) H7, N9 and M genes by real-time RT-PCR.

**Figure 2:**
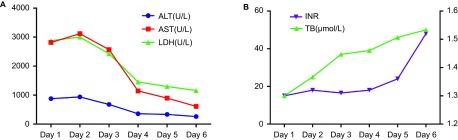
Dynamic changes in biochemical test results in case 1. (**A**) Changes in ALT, AST and LDH values during the hospital stay. (**B**) Changes in TB and INR values during the hospital stay. ALT, alanine aminotransferase; AST, aspartate aminotransferase; INR, international normalized ratio; LDH, lactate dehydrogenase; TB, total bilirubin.

On admission, severe liver injury was identified. Serum ALT, aspartate aminotransferase (AST) and lactate dehydrogenase (LDH) activities were elevated to 878, 2814 and 2879 U/L, respectively. Total bilirubin level was 25 μmol/L. The international normalized ratio (INR) was prolonged to 1.3 with a D-dimer value of 12970 μg/L. The dynamic changes in liver function are shown in Figure 2. Peak ALT, AST and LDH activities (937, 3117 and 3001 U/L, respectively) occurred on the second day of admission, while the INR peaked at 1.52 on the sixth day. Renal failure was identified as the creatinine level reached 314 µmol/L with anuria. Serologic test results were negative for viral hepatitis A, B, C, D, E, Epstein–Barr virus and cytomegalovirus. Serum autoantibodies were all negative. Liver ultrasound revealed a dilated hepatic vein (left branch, 1.22 cm; middle branch, 0.61 cm; and right branch, 1.36 cm) with slow hepatic vein flow (middle branch, 9.14 mm/s) (Figure 1B and 1C) and excluded surgical biliary tract diseases and space-occupying lesions. An echocardiogram showed a dilated left ventricle with an internal diameter at end-diastole (LVIDd) of 56.46 mm and low left ventricular ejection fraction (LVEF) of 42% with weakened left ventricular wall motion (Figure 1D). The patient had a 20-year history of hypertension and coronary artery disease status and had twice undergone placement of intracoronary stents at six and two years previously. Concomitant acute myocardial infarction was excluded according to the echocardiogram, electrocardiogram (ECG), myocardial enzyme spectrum and troponin (TNI) results.

The patient was identified as having a severe A(H7N9) infection with MOF and HH, placed on a mechanical ventilator and given fluid resuscitation. Antiviral therapy with oral osetalmavir (75 mg) twice daily was administered through a feeding tube. Plasma exchange and haemofiltration were performed in the patient as liver and renal replacement therapies.

**Figure 3:**
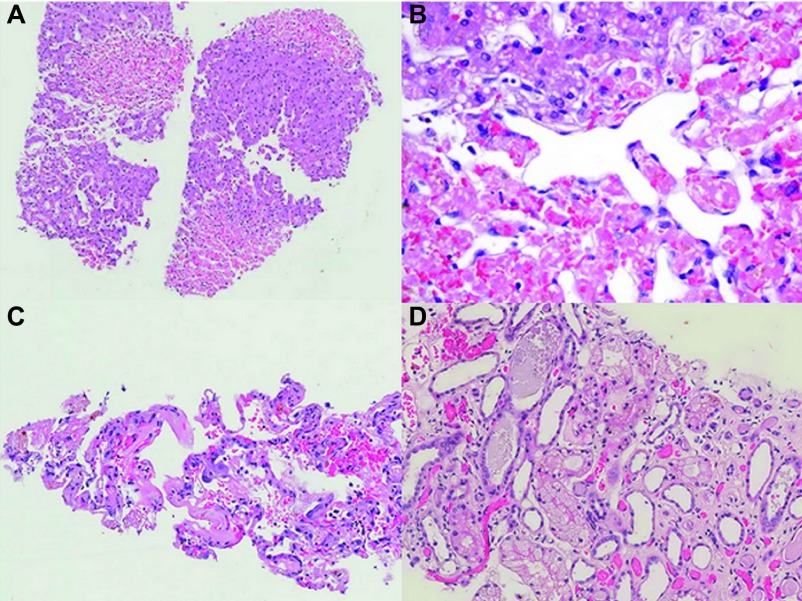
Histological findings in case 1. (**A**) Centrilobular necrosis in the liver without inflammation (haematoxylin and eosin, 10×). (**B**) Dilated sinusoids with coagulative necrosis (haematoxylin and eosin, 40×). (**C**) Reactive type II pneumocytes and hyaline membrane in the lung (haematoxylin and eosin, 20×). (**D**) Renal tubular degeneration and necrosis (haematoxylin and eosin, 20×).

Unfortunately, the patient died on day 6 of hospitalization. The liver biopsy showed well-demarcated multifocal centrilobular coagulative necrosis without accompanying inflammation. The necrotic hepatocytes were partially replaced by red blood cells, outlined by sinusoidal endothelial cells. Sinusoid congestion and mild hepatocellular atrophy were identified in adjacent tissue (Figure 3A and 3B). The lung showed interstitial pneumonitis with hyaline membranes, congestion, intrapulmonary haemorrhage and anthracosis (Figure 3C). Acute tubular necrosis and generalized renal tubule atrophy were found in the kidney (Figure 3D).

### Case 2

A 58-year-old male was admitted to the ICU with a two-week history of cough with sputum and sudden deterioration with a fever of 38.1 °C prior to admission. On admission, the patient had paroxysmal ventricular tachycardia, and his mean arterial pressure was maintained at 70 mmHg by administration of norepinephrine at a dose of 0.67 μg/kg·min. Oxygen inhalation through a nasal tube was administered to maintain oxygen saturation at >90%.

**Figure 4:**
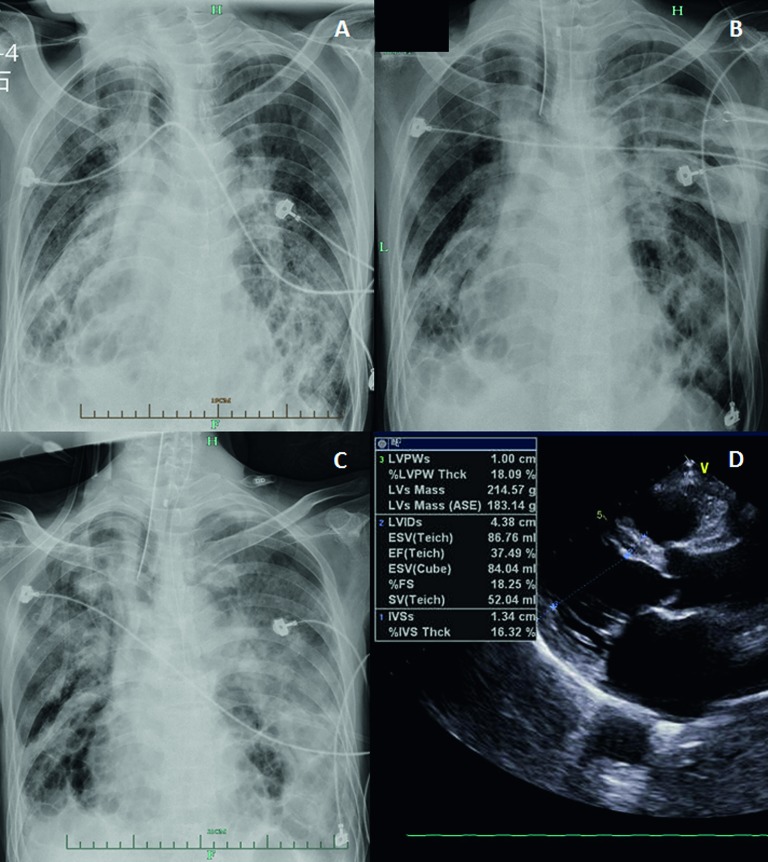
Radiographic and ultrasound findings in case 2. (**A**) Chest radiograph at the time of admission showed bilateral pulmonary infiltration. (**B**) Chest radiograph on day 2 compared with that at admission showed progression of the bilateral pulmonary infiltration and consolidation. (**C**) Chest radiograph on day 4 showed progression of bilateral pulmonary infiltration and consolidation compared with day 2. (**D**) Echocardiogram at the time of admission showed cardiac failure (LVEF 37.5%). LVEF, left ventricular ejection fraction.

The patient had a recent history of contact with live poultry 10 days prior to admission. Respiratory secretions were positive for A(H7N9) H7, N9 and M genes. Chest radiography on admission showed bilateral pulmonary infiltrates (Figure 4A). On admission, an echocardiogram showed a dilated left ventricle with a low LVEF of 37.5% (Figure 4D), together with weakened cardiac wall motion. The serum creatine phosphokinase (CK) level was 799 U/L, and TNI was negative. ECG did not show the evidence of acute myocardial infarction on admission. A second echocardiogram showed recovery of LVEF to 62% without accompanying weakened cardiac wall motion at day 3. The patient had a 30-year history of bronchiectasia that had been progressing to pulmonary heart disease for 10 years.

**Figure 5:**
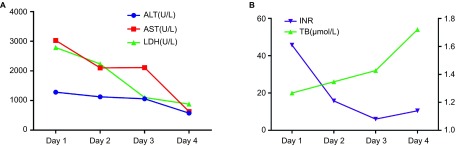
Dynamic changes in biochemical test results in case 2. (**A**) Changes in ALT, AST and LDH values during the hospital stay. (**B**) Changes in TB and INR values during the hospital stay. ALT, alanine aminotransferase; AST, aspartate aminotransferase; INR, international normalized ratio; LDH, lactate dehydrogenase; TB, total bilirubin.

On admission, severe liver injury was identified. ALT, AST and LDH activities were elevated to 1281, 3029, and 2787 U/L, respectively, the highest levels seen during the patient's hospital stay (Figure 5). Total bilirubin level was 20 μmol/L. INR was prolonged to 1.61 with a D-dimer value of 57872 μg/L. The dynamic changes in liver function and INR are described in Figure 5.

The patient was identified as having a severe A(H7N9) infection with MOF and HH. Antiviral therapy of oral oseltamavir (75 mg) twice daily was administered. Due to the progression of the infiltrates and consolidation (Figure 4B and 4C), the patient was placed on a mechanical ventilator on the third day after admission. Continuous haemofiltration was given when the creatinine level reached 135 µmol/L with oliguria on the third day of admission. Unfortunately, the patient died on day 4 of hospitalization due to deterioration of MOF.

## Discussion

A(H7N9) infection in humans was first identified in February 2013.^1^ It was described as a flu-like illness that could lead to respiratory failure and be accompanied by injuries to other organs, such as the liver, kidney, heart, etc.^5,9,15,16^ According to early reports, the occurrence of liver injury in A(H7N9) patients was relatively frequent.^5,15^ Our study is not only in agreement with these reports, but it also indicated a higher frequency of liver impairment during the hospital day (*n* = 51, 45.5%) than on admission (*n* = 27, 24.1%) (*P* < 0.001). However, severe liver impairment (ALT > 20-fold the ULN) was rare (*n* = 2, 1.8%). The distinct difference between the extent of elevated ALT between HH patients and non-HH patients with liver impairment indicated that HH could be differentiated from other types of liver injury by means of a sharp elevation in the ALT level. The majority of A(H7N9)-infected patients are treated with antiviral medications, antibiotics and steroids, which are potentially hepatotoxic.^17,18,19,20^ There are also reports of liver impairment induced by respiratory viruses, such as severe adult respiratory syndrome coronary virus (SARS-CoV).^17^ Hence, it is meaningful to identify the cause of liver injury, especially in cases of severe liver impairment.

HH is a common cause of acute liver injury in the ICU setting and is characterized by an abrupt and massive increase in aminotransferase activity secondary to anoxic centrilobular liver necrosis.^7,8^ In other words, it is the clinical syndrome underlying hepatic necrosis homogeneously distributed around the central veins. Centrilobular liver necrosis without inflammation was found in this study, which is consistent with the typical histological changes of HH (Figure 3A and 3B).^7,21^ The pathologic features differed from those of SARS-CoV-associated viral hepatitis, in which hepatocyte ballooning and lobular lymphocytic infiltration are commonly found.^17^ The negative results of RT-PCR testing of post-mortem liver tissue excluded A(H7N9)-related viral hepatitis. In addition, the peak LDH activities of 3001 and 2879 U/L in these patients were notable (Figures 2A and 5A). These may be useful for differentiation of HH from viral hepatitis.^22^

According to previous reports, the occurrence of HH requires a pre-existing condition that chronically compromises oxygen supply to the liver, together with an acute event that further decreases hepatic oxygen supply.^10,13^ Severe A(H7N9) infection will lead to respiratory failure, and respiratory failure will in turn induce a sudden decrease in oxygen supply to the liver.^9^ The presence of a chronic disease that reduces baseline oxygen supply to the liver is an indicator of HH risk in A(H7N9) patients. In case 1, coronary artery disease that chronically reduced blood flow, and hence oxygen supply, to the liver was found in both HH cases. In case 2, chronic pulmonary dysfunction decreased blood oxygen saturation, which led to an insufficient baseline oxygen supply to the liver. In addition, temporary left ventricular failure and respiratory failure decreased the oxygen supply to the liver. Temporary left ventricular failure was suspected due to myocarditis. The CK, TNI and ECG results supported this speculation. In another temporary left ventricular failure patient with A(H7N9) infection, typical myocarditis histological changes, such as cluster lymphocyte infiltration, were found in post-mortem heart tissue. Cardiac dysfunction was confirmed on cardiogram by the decrease in LVEF to 42% and 37.5% in the two patients on admission. In case 1, the detection of dilated intrahepatic veins by abdominal ultrasound (Figure 1B) and a prolonged history of coronary disease with two intracoronary stent implantation procedures suggested chronic right ventricular failure. During hospitalization, these two patients required persistent intravenous norepinephrine transfusion to maintain diastolic blood pressure. This clearly indicated circulatory failure, which would also reduce oxygen supply to the liver. The reduction in oxygen supply caused by respiratory failure was ameliorated in the ICU. The peak ALT and AST levels occurred on the day of admission or the following day in this study, which is in accordance with the report of Raurich *et al*.^12^

Ours is the largest centre for the treatment of A(H7N9) patients in the world. A(H7N9) patients in our ICU represent ∼20% (112/571) of the total number of patients confirmed infected with A(H7N9) according to the latest report by the WHO.^2^ The prevalence of HH in ICU patients with A(H7N9) infection in our centre is 1.8%. The incidence of HH in ICU patients varies from 0.9% to 11.9%.^7^ Most studies have reported a prevalence of 1%–2%. This is in agreement with our findings, which suggest that patients with A(H7N9) infection in the ICU setting have a similar prevalence of HH to that of those with other aetiologies.

The in-hospital mortality rate of HH is ≥45%.^7^ According to Raurich *et al.*, risk factors for mortality include prolonged INR and need for renal replacement.^12,23^ Both of these risk factors were present in the two patients reported herein, which was indicative of a poor prognosis. Both patients died despite treatment of the accompanying MOF (such as by fluid resuscitation and blood purification).

According to previous reports, a cytokine storm can occur in severe A(H7N9) or A(H5N1) patients.^14,24,25^ Moreover, inflammatory cytokines were found to contribute to hypoxic hepatopathy in animal models.^26^ A cytokine storm was also detected in two HH patients with A(H7N9) infection. However, the relatively low prevalence of HH (2 HH cases vs. 110 non-HH cases) makes it difficult to reach a conclusion regarding the contribution of a cytokine storm to the pathogenesis of A(H7N9) infection in HH. A large-scale study of A(H7N9)-infected patients should be performed to determine the contribution of a cytokine storm to HH and the correlation between them.

In conclusion, we report here a 1.8% prevalence of HH in ICU patients with A(H7N9) infection. The typical pathological change of HH, centrilobular necrosis of the liver, was confirmed. A(H7N9)-related viral hepatitis was excluded based on negative real-time RT-PCR for viral genes in liver tissue. A lower LVEF accompanying underlying chronic heart disease and abrupt elevation of serum ALT levels at the time of admission in patients with A(H7N9) infection suggest the need for identification and treatment of HH.^8^

## Figures and Tables

**Table 1:** Primers and probes used for avian influenza A(H7N9) virus RT-PCR testing in this study

Gene	Primer	Probe
H7	forward AGAGTCATTRCARAATAGAATACAGAT reverse CACYGCATGTTTCCATTCTT	FAM-AAACATGATGCCCCGAAGCTAAAC-BHQ1
N9	forward GTTCTATGCTCTCAGCCAAGG	HEX-TAAGCTRGCCACTATCATCACCRCC-BHQ1
	reverse CTTGACCACCCAATGCATTC	
M	forward GAGTGGCTAAAGACAAGACCAATC	FAM-TCACCGTGCCCAGTGAGCGAG-BHQ1
	reverse TTGGACAAAGCGTCTACGC	

**Table 2:** Clinical features of the patients with A(H7N9) infection (*n* = 112)

**Age**	**Number (%)**		
≤14 years	0 (0)		
>14 – ≤65 years	78 (69.6)		
>65 years	34 (30.4)		
**Sex**			
Male	75 (67.0)		
Female	37 (33.0)		
**Underlying medical conditions**			
Hypertension	54 (48.2)		
Coronary heart disease	12 (10.7)		
Chronic obstructive pulmonary disease	6 (5.4)		
Cerebrovascular disease	5 (4.5)		
Chronic liver disease	7 (6.3)		
Chronic renal disease	4 (3.6)		
Diabetes mellitus	17 (15.2)		
Rheumatoid arthritis	3 (2.7)		
Cancer	7 (6.3)		
**Liver impairment**		**Value (mean**±**SD) U/L**	
** On admission**	27 (24.1)	156.42±282.64	
ULN < ALT ≤ 20-fold ULN	25 (22.3)	79.50±56.34	
ALT > 20-fold ULN	2 (1.8)	1079.50±41.72#	
** During hospitality day**	51 (45.5)*	142.35±217.21	
ULN < ALT ≤ 20-fold ULN	49 (43.8)	102.90±86.11	
ALT > 20-fold ULN	2 (1.8)	1109.00±243.24†	
**Antiviral therapy**	112 (100)		
**Antibiotic therapy**	91 (81.3)		
**Glucocorticoid therapy**	63 (56.2)		

* liver impairment ratio (during hospital stay vs. on admission, *P* < 0.001)

# ALT level on admission (HH patients vs. liver injury without HH, *P* < 0.0001)

† peak ALT level during hospital stay (HH patients vs. liver injury without HH, *P* < 0.0001)

acute respiratory distress syndrome, ARDS; alanine aminotransferase, ALT; aspartate aminotransferase, AST; lactate dehydrogenase, LDH; upper limit of normal, ULN; standard deviation, SD; hypoxic hepatitis, HH.
