# Author Correction: Prevalence and characteristics of hypoxic hepatitis in the largest single-centre cohort of avian influenza A(H7N9) virus-infected patients with severe liver impairment in the intensive care unit

**DOI:** 10.1038/s41426-018-0054-9

**Published:** 2018-03-29

**Authors:** Yi Min Zhang, Ji Min Liu, Liang Yu, Ning Zhou, Wei Ding, Shu Fa Zheng, Ding Shi, Lan Juan Li

**Affiliations:** 1 State Key Laboratory for Diagnosis and Treatment of Infectious Diseases, Collaborative Innovation Center for Diagnosis and Treatment of Infectious Diseases, the First Affiliated Hospital, College of Medicine, Zhejiang University, 310003 Hangzhou, Zhejiang Province China; 2 Department of Pathology and Molecular Medicine, Faculty of Health Sciences, McMaster University, Hamilton, L8S 4L8 ON Canada; 3 Department of Pathology, the First Affiliated Hospital, College of Medicine, Zhejiang University, 310003 Hangzhou, Zhejiang Province China

**Correction to:** *Emerging Microbes & Infections* (2016)** 5:**e1; 10.1038/emi.2016.1; Article published online 6 January 2016

The authors apologized for an error in the first affiliation. The original research summary statement “H7N9 influenza-infected patients with chronic heart disease accompanying acute heart failure are at elevated risk of severe liver damage. Lan-Juan Li and colleagues at Zhejiang University in Hangzhou studied 112 patients admitted to intensive care with H7N9 influenza over 2 years, two of whom had hypoxic hepatitis, a type of acute severe liver injury caused by reduced oxygen supply to the liver. The results indicated a combination of pre-existing heart conditions and sudden decreasing of left ventricular ejection fraction in the two patients, combined with the respiratory failure caused by H7N9, reduced oxygen supply to the liver, which caused *hereditary hemochromatosis*. The researchers suggest that H7N9 patients with a history of heart disease and acute left heart failure on admission should be carefully checked for liver damage.” should be changed to “H7N9 influenza-infected patients with chronic heart disease accompanying acute heart failure are at elevated risk of severe liver damage. Lan-Juan Li and colleagues at Zhejiang University in Hangzhou studied 112 patients admitted to intensive care with H7N9 influenza over 2 years, two of whom had hypoxic hepatitis, a type of acute severe liver injury caused by reduced oxygen supply to the liver. The results indicated a combination of pre-existing heart conditions and sudden decreasing of left ventricular ejection fraction in the two patients, combined with the respiratory failure caused by H7N9, reduced oxygen supply to the liver, which caused *hypoxic hepatitis*. The researchers suggest that H7N9 patients with a history of heart disease and acute left heart failure on admission should be carefully checked for liver damage.”

